# Supplementary material for: High-Throughput Synthesis of Nanogap-Rich Gold Nanoshells Using Dual-Channel Infusion System
Source: Int J Mol Sci. 2024 Jan 29;25(3):1649. doi: 10.3390/ijms25031649 (PMC10855030; doi:10.3390/ijms25031649)
Supplement: Supplementary file 1 [file ijms-25-01649-s001.zip › ijms-2808563-supplementary.pdf]

High-Throughput Synthesis of Nanogap-Rich Gold Nanoshells Using Dual-Channel Infusion System

Yoon-Hee Kim [1], Hye-Seong Cho [1], Kwanghee Yoo [1], Kyeong-Min Ham [1], Homan Kang [2], Xuan-Hung Pham [1,*] and Bong-Hyun Jun [1,*]

[1] Department of Bioscience and Biotechnology, Konkuk University, Seoul 05029, Republic of Korea; yoonhees@konkuk.ac.kr (Y.-H. Kim); joh0302@naver.com (H.-S. Cho); heu1997@konkuk.ac.kr (K. Yoo); hkm7321@konkuk.ac.kr (K.-M. Ham)

[2] Gordon Center for Medical Imaging, Department of Radiology, Massachusetts General Hospital and Harvard Medical School, Boston, MA 02114, USA; hkang7@mgh.harvard.edu (H. Kang)

* Correspondence: phamricky@gmail.com (X.-H. Pham); bjun@konkuk.ac.kr (B.-H. Jun)

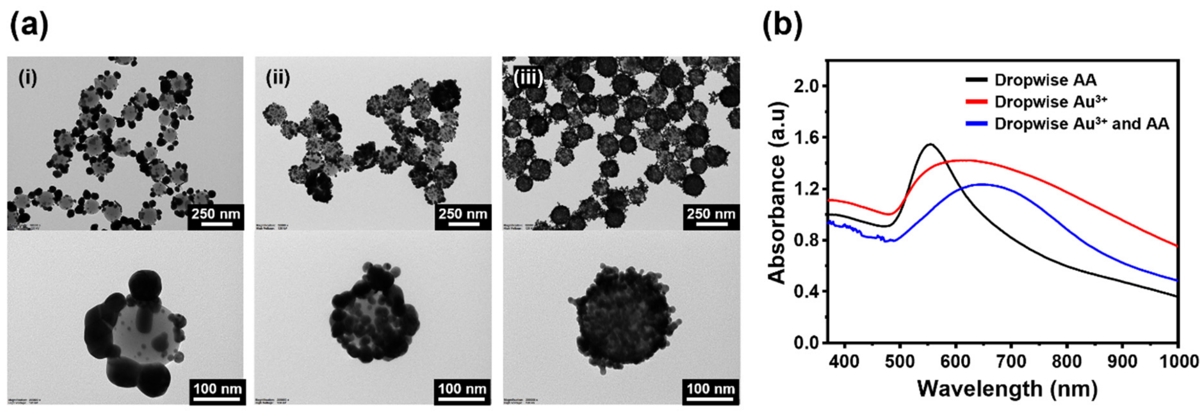

Figure S1. (**a**) TEM images and (**b**) UV-visible absorption spectra of SiO_2@Au NS prepared by controlling the addition of Au^{3+} precursor and AA solution: (i) dropwise addition of AA solution after batch addition of Au^{3+} solution, (ii) dropwise addition of Au^{3+} solution after batch addition of AA solution, and (iii) dropwise addition of both Au^{3+} and AA solution.

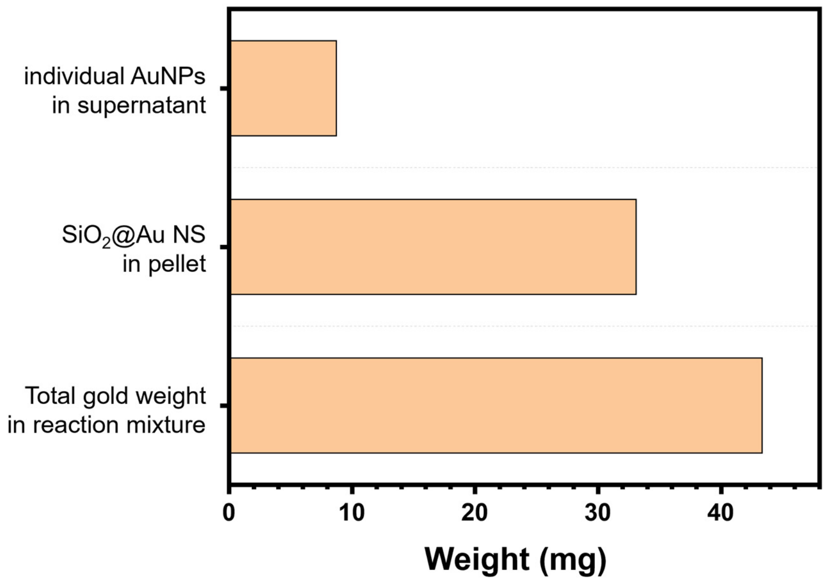

Figure S2. Gravimetric analysis of individual Au NPs and grown SiO_2@Au NS in the reaction mixture of high-throughput seed-mediated growth process with 4.4 mL Au^{3+} precursor solution. The supernatant (containing individual AuNPs) and pellet (containing SiO_2@Au NS) were separated by centrifugation with 800 g. The increased

weight of each component was measured after drying solvent in vacuum chamber.

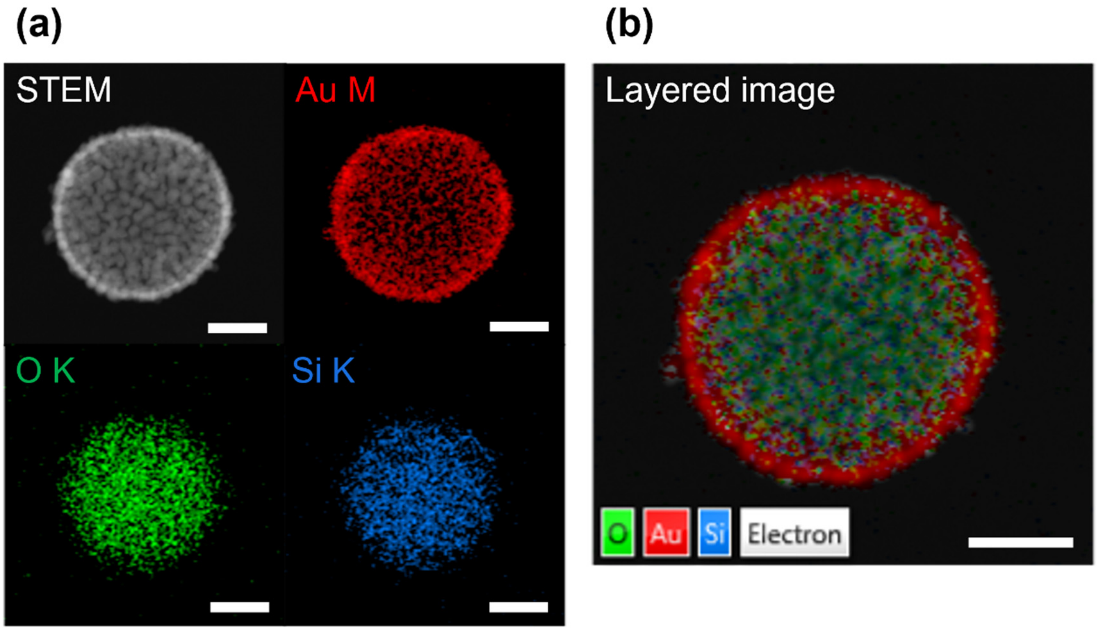

Figure S3. EDS maps of high-throughput synthesized SiO_2@Au NS captured by Cs-corrected TEM (JEM-ARM200F, Jeol, Tokyo). (**a**) Scanning transmission microscope image and EDS mapping images of gold (red dots), oxygen (green dots) and silicon (blue dots). (**b**) STEM-EDS layered mapping image of SiO_2@Au NS. Inset scale bars indicate 50 nm.

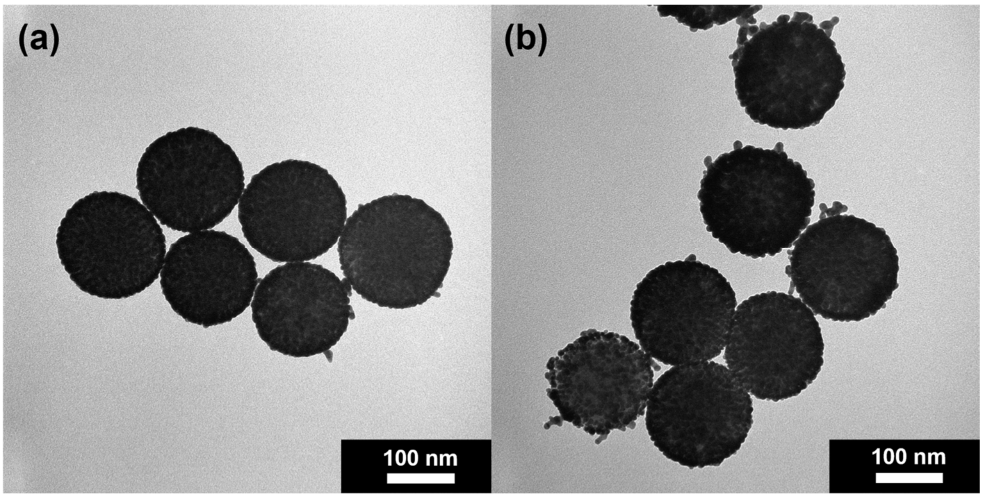

Figure S4. TEM images of SiO_2@Au NS prepared through (**a**) the reported 0.2 mg NPs scaled method and (**b**) high-throughput 10 mg NPs scaled method (this work).
